# Report on the Causes Which Impede the Progress of American Medical Literature

**Published:** 1857-05

**Authors:** 


ART. IV.—*Report on the Causes which impede the Progress of American Medical Literature.* By S. D. GROSS, M.D. From the Transactions of the American Medical Association, Vol. IX, 1856.

As the annual volumes of Transactions published by the American Medical Association have but a very limited circulation, too limited, we are sorry to say, for the good which they are calculated to accomplish, we propose to give, from time to time, analyses of some of the more important papers which they contain; and as no one of the contributions to the last volume has attracted more general attention, and provoked more various criticism than the "Report" of Dr. Gross, we do not doubt that the readers of the "Review" will thank us for presenting them with an abstract of its contents. The sentiments expressed by the author, and the conclusions which he has reached, mortifying on some accounts as they are to our national pride, commend themselves to the careful, conscientious, and temperate consideration of all who have at heart the high interests of our profession. The stigma, which is so often cast upon us by our transatlantic brethren, is not to be removed by the amiable listlessness of old fogies, who sit quietly around the professional fire-places of their ancestors, nor by the empty boastings of the self-styled Young Physic, but by a close and scrutinizing self-examination, a determination to know the truth, and a hearty acquiescence in every rational plan of progress. However much men may differ in reference to the points supposed to be established by the author, he deserves our lasting gratitude for his open and manly attempt to point out the evils under which we labor; and the severe rubbing which he has given the thin-skinned portions of the *body* of the profession, will doubtless produce a healthier action, notwithstanding the writhings and revilings which some individuals, smarting with well-deserved pain, have indulged in. It is not our intention here to defend the author from the violent criticism based upon misinterpretation, and wilful misrepresentation with which his report has been met in certain quarters, where we had a right to expect a more candid and high-minded spirit, while from the disgusting personalities, with which one or two so-called medical periodicals have seen fit to soil their pages, his position as a gentleman is a sufficient shield. Our object is simply to place the substance of the Report before the readers of the Review, that they may judge for themselves.

After stating the peculiar difficulties with which the subject is environed, the Report proceeds immediately to consider the nature and extent of our home medical literature. In this connection the avowal is frankly made, that, notwithstanding the rapid strides that the profession has taken within the past few years, our medical literature, though highly creditable as far as it goes, is still in a state of infancy, imperfect in many of its parts, and struggling against obstacles—a fact which the Association necessarily admitted in appointing the committee. In explanation of this state of things, reference is made to the comparative youthfulness of our national existence; our recent entire dependence upon European supplies, a dependence which is still more or less acknowledged by many; the slowness with which all branches of literary pursuit necessarily proceed toward maturity; and the absence of anything like governmental aid or patronage. It is consoling to observe, however, that the development of our national medical literature has been comparatively more rapid than that of our mother country, or any of her continental neighbors; for, as here mentioned,—

"Ages elapsed before England, France, Germany, and Italy, the most favored, learned, and scientific nations of the Old World, had made even a beginning in medical authorship. The medical literature of Great Britain dates no further back than the time of Harvey and Sydenham, in the early part of the seventeenth century. Until the appearance of those illustrious men, the pride and glory of English medicine, England had no medical writers who survived the generations of which they formed a part; and even for a long time subsequently, she hardly produced a solitary work which is now remembered except by its title. Richard Wiseman was her first great surgical author, and generations passed away before she produced a Pott and a Hunter. In anatomy, physiology, chemistry, materia medica, medical jurisprudence, toxicology, obstetrics, and practical medicine, she had literally no great works until the commencement of the present century. In pathological anatomy, and general pathology, she has not yet a solitary treatise worthy of her noble profession. The same is true, though not to an equal degree, of the medical literature of some of the other nations of Europe."

During our colonial dependency, owing to the peculiar difficulties connected with a settlement of a wide-extended country, the sparseness of the population, the few inducements for the emigration of refined and educated physicians from Europe, the lack of medical schools, and the great expense attending a foreign education, the medical profession was almost necessarily composed of men of comparatively slender acquirements, while the clerical and legal professions, especially the former, were much more ably represented.

"The religious persecutions of Europe induced some of the most able and learned divines to forsake the Old World to seek an asylum and a home in the New. They brought with them their piety, their zeal, their erudition, and their enterprise, which they devoted, without stint, without money, and without price, to the service of the Church, of education, and of literature. They became the founders of some of our most valuable and distinguished literary institutions, and the authors of eminently creditable works on theology and history. The most important judicial offices were held by men of learning and legal acumen, sent hither by the British Crown; and many of the governors of the different provinces were Englishmen distinguished for their literary and scientific tastes and attainments."

But how different was it with the profession of Medicine; she had no teachers worthy of the name, no antecedents, no great lights, no influence.

"The first American Medical College was erected in 1763, and although another was soon added, yet both were compelled to close their halls during the Revolution; nor was any attempt made to revive them until after the establishment of peace in 1783. Up to this period, and indeed till after the commencement of the present century, hardly any work had appeared on medicine from the pen of a native physician. The most valuable treatises then extant were Bard's *Midwifery* and Jones's *Surgery*, both greatly esteemed in their day on account of their practical character."

Under the genial influences, however, of freedom and self-reliance, medical societies, medical charities, and, finally, medical periodicals, sprang up in every direction, and afforded employment and labor for the best intellects of the day. As a necessary result, book after book has appeared, until we shall soon cease to be able to count them.

"The early part of the present century supplied us with the writings of Rush and Barton, the *System of Anatomy*, by Wistar, Dorsey's *Surgery*, so long used as a text-book in the University of Edinburgh, Chapman's *Therapeutics and Materia Medica*, Coxe's *Dispensatory*, Thatcher's *Practice of Medicine*, and several other productions of minor note. As yet, however, the profession had not produced one solitary great work on any subject. Then appeared, in pretty rapid succession, the valuable treatises of T. R. Beck, Gibson, Dewees, Horner, Eberle, Hare, Silliman; and, at a still later period, those of Dunglison, Wood and Bache, N. R. Smith, Meigs, Wood, Dickson, Oliver, Paine, Condie, Bell, Warren, Stewart, Ray, Gerhard, Bartlett, McClellan, Pancoast, Morton, Miller, Mitchell, Frost,

H. H. Smith, and others. The last few years have been unusually prolific in valuable monographs, as is exemplified by the publications of the younger Meigs, the two Stillés, Swett, Carnochan, Frick, Drake, and La Roche, the last two of which may justly be regarded as forming an epoch in the literature of the profession."

After thus proving that the profession of the United States at the present day cannot justly be accused of idleness or ignorance, but that its aggregate capacity comprises an amount of talent, erudition, science, and activity, greater than that of any of the other liberal professions, the Report proceeds to inquire into the causes which still obstruct the progress of our national medical literature. These are grouped under four principal heads: "1. The identity of the language of this country with that of Great Britain. 2. A disposition in the profession to patronize English works in preference to American. 3. A want of independence in our periodical press. 4. A lack of industry in observing and recording facts in private and hospital practice."

The argument upon the first head is based upon the easy accessibility of foreign works written in our own langnage, and the free use that is made of them by our large publishing houses to the exclusion of native productions. We have the testimony of the English journals that when a new book upon almost any branch of medicine is announced in London or Edinburgh, the agents of some of our American houses are placed upon the look-out, and no sooner do the first copies drop from the press, than one or more are seized upon and whisked across the sea as rapidly as steam can carry them, and before the original work reaches us in the usual course, its American reprint is laid upon our table for notice. This, it is true, is a regular business transaction, and in the absence of an international copyright law, perfectly legitimate; but its injury to our home literature is not, on this account, any the less serious. It may be argued, on the contrary, that the successful sale of these republications is an evidence that the wants of the profession are thus acknowledged and supplied. But when has it been proved that where a man can't get home-made bread he will not send to the baker? And if this matter is to be looked at in a purely mercantile sense, why may not government impose a duty upon such importations as well as upon other foreign articles which do not come under the head of necessaries? And no one will contend, we presume, that foreign books are in any sense more necessary to our comfort than many other articles of merchandise. But if it be true that the supply of republications is only commensurate with the demand, then the difficulty is expressed in the premises under the second head, to wit, a disposition on the part of the profession to patronize British works in preference to American. But this argument from the demand to the supply, should be viewed in another light, for the American publisher being exempt from the expense of a copyright, is enabled to furnish the reprints at a comparatively low price, and thus appeals to the pecuniary interests of the profession; an influence which, however vehemently it may be denied when thus broadly stated, is silently but surely at work in keeping the medical mind of this country in more or less subjection to British authority. And thus it is, as stated in the Report, that "one of the most remarkable anomalies of our age and country that our literature, polite as well as scientific, exhibits, even at the present moment, more of a foreign than a domestic aspect."

"It cannot be denied that this practice of republication is of great advantage to the public; for it serves to diffuse among the people, in a cheap and accessible form, a vast amount of knowledge that would otherwise be beyond their reach. It brings the works of our transatlantic brethren directly to our doors, at the same time that it serves to extend the name and fame of the authors. But, while it accomplishes all this, it unfortunately interferes, in the most positive manner, with the establishment of our national literature, considered in its widest as well as in its professional sense. It depresses native talent, native genius, native aspiration and enterprise. It narrows the road of authorship, and besets it with obstacles and difficulties almost insurmountable by ordinary means. The man who attempts to scale it must do so at the risk of a long probation, without the prospect even of much ultimate pecuniary reward, when his reputation shall have been firmly established by the product of his pen. When his literary labors are completed, and he places his MS. into the hands of his publisher, should he be so fortunate as to find one, he is pretty sure to be informed that there are already before the public so many works on the same subject as to render the success of his own too uncertain to justify the offer of a stipulated sum for the copyright of the first edition. Should the sale go off well, he may ultimately receive a few hundred dollars for what ought to have brought so many thousand, if the book had no foreign competitors in the form of reprints. In short, he finds his only recompense and solace in the prospective fame of his literary labors. I do not now speak of the novelist, the poet, the tourist, and other writers of light literature, who always find readers, however trashy their productions, but of men of science and letters, the solid and substantial authors of a country. These men must be encouraged, patronized, and sustained in their efforts to improve the literature of the nation, and thus advance its honor and glory. If it be said, in reply to these remarks, that every work must stand on its own merits, we do not perceive the force of the rejoinder. If the market is already glutted with foreign productions, it must be obvious that the wants of the public are, at least, in part, if not fully supplied; and hence the native work, however meritorious, must labor under the disadvantage of a degree of competition, which, especially if we consider the additional disadvantage of its higher price, may throw it into the shade, or altogether prevent its success. If, for instance, an American physician were to write a work on surgery, his success would be sure to be tardy, because the country is already flooded with foreign treatises; and so in regard to most of the other departments of the profession, as well as of literature in general. If the people of Europe spoke but one language, and there existed no international copyright laws, there would be comparatively few authors of any kind; the necessity for writing would be much diminished; the same book would answer for all; and men of literary taste and attainment would seek other channels for the exercise of their talents. But such is not the fact. Every nation has a distinct and separate language; a distinct and separate literature. The works of Sydenham and Harvey, of Van Swieten, Bichat, Haller, Baglivi, and Scarpa can be read out of their own countries by the mass of the profession only through the medium of a translation. Speaking, as we do, the same language as the people of Great Britain, and republishing as we do, at pleasure, any of their works that come within our reach, Shakspeare has more readers by ten to one on the shores of the Ohio than on the banks of the Avon."

In the discussion of the second point, viz.: "The disposition of the profession to patronize foreign works, especially English, in preference to our own," the Report sets forth a variety of circumstances under which this disposition is exhibited. It is stated in the first place,—and who can deny it?—that the majority, or at least a large proportion of the text-books recommended by the various medical schools in this country, are of English origin, while every one must be aware that upon most of the branches taught in these schools, there are native treatises not inferior to the foreign. In a brief comparison between some of the medical authors of the United States and those of Great Britain the following paragraph occurs:

"Where is there, it may be asked, in the English language, a medical dictionary at all comparable with that of Professor Dunglison? or a treatise on physiology superior to that of the same distinguished author? Since the days of Haller, I have hardly seen a more learned, systematic, or comprehensive treatise on the subject in any language, certainly not in the English. It is all that such a work should be for the pupil and practitioner; plain, simple, perspicuous, and perfectly methodical, with an amount of erudition as rare as it is profound and astonishing. Our works on anatomy are amply sufficient for all the purposes of the class-room, for which they have all been employed, to a greater or less extent, by some of our schools. The system of Wistar has maintained its place in the esteem and affection of the American student for nearly half a century, and, with the emendations and additions of Professor Pancoast, bids fair to hold out half a century longer. Horner's treatise has passed through numerous editions; and the works of Morton, Richardson, and Handy, are, in every respect, superior to that of Wilson, which figures so conspicuously upon the catalogues and annual announcements of our colleges. In practical medicine we have just cause to be proud of the labors of Eberle, Wood, Dunglison, and Dickson, to say nothing of those of Hosack, Dewees, Thatcher, and Bell, the latter of which, however, is too much mixed up with that of Dr. Stokes to give it a national air. In materia medica and therapeutics, the treatises of Chapman, Eberle, and Dunglison, have long been held in the highest esteem. The second of these works was honored, soon after its appearance, with a German translation, and its author with a membership of the Medico-Chirurgical Society of Berlin. The treatise of the late Dr.

John B. Beck, a more recent production than any of the above, is, I conceive, a model for a text-book; clear, graphic, concise, yet sufficiently comprehensive for all possible purposes to which such a book can be applied. In obstetrics we have the admirable and original works of Dewees, Meigs, and Miller, which all enjoy a European reputation, and a wide appreciation at home. On the diseases of children, the treatises of Eberle, Condie, Stewart, and Meigs are without rivals in the English language. In medical jurisprudence there was no work, until recently, that was at all equal to that of T. R. Beck, the merits of which have been acknowledged even in Great Britain by several reprints, and in Germany, by at least one translation. The recent production of the late lamented Moreton Stillé, on the same subject, is destined to attain a high rank in the medical literature of the country. The Dispensatory of Wood and Bache is, beyond doubt, the most able work of the kind extant. If we have no great treatises on surgery, chemistry, toxicology, and some other subjects, is it to be supposed that we are incapable of supplying them? Certainly not. What has been done for the other departments of medicine may assuredly be done for these. We have the power; it is only necessary to exert it. Ages elapsed before Great Britain produced one solitary great work on surgery, obstetrics, practical medicine, toxicology, chemistry, medical jurisprudence, and anatomy. The Universities of Oxford and Cambridge had existed for centuries before she even began to dream of a national medical literature. She has not even yet anything like a great medical and surgical dictionary, one to be compared, in point of extent and erudition, with those of France and Germany. In descriptive anatomy, until the appearance of the treatise of Mr. Quain, only a few years ago, she had never furnished one great, respectable, or reliable work; and in materia medica, toxicology, juridical medicine, and obstetrics, she was equally destitute. In operative surgery she has produced nothing equal to the elaborate and beautiful work of Dr. Pancoast, with, perhaps, the single exception of that of Mr. Fergusson, so well known and so highly appreciated in this country."

The reporter, however, disclaims the least desire to see an embargo laid upon foreign works,—

"Or in any manner, form, or degree, disparage their merits, or discountenance their just claims upon the favor and patronage of the American profession. Literature, the arts and sciences, are cosmopolitan, acknowleding no 'pent up Utica' as their home. Like the breezes of heaven, which fan and fertilize the earth, and refresh its laborers, they belong to no country and to no age, but to the whole world and to all time. We need no international copyright laws; let there be a free interchange of our intellectual products; let us not place upon them any restrictions, as we do upon calico and other articles of manufacture. The time will come, if it has not already come, when the medical profession of Great Britain will be glad to receive, in par exchange, the results of our mental labor for theirs."

In this connection allusion is also made to the editing of foreign works by American physicians, and the practice condemned upon the broad ground that these republications should be made to rest upon their own merits. It is well known that in many instances such works, so far from being improved by the addition of American notes, are intrinsically damaged, although the scribblings of the editors may increase their sale, while those that are much improved are generally not worth reprinting, and should not therefore be foisted upon the profession in this way. That this practice, so humiliating to our national pride and opposed to every feeling of patriotism, has met with but little reciprocity in England, is proved by the fact that the only American works that have ever been republished in that country, are Dorsey's *Surgery*, Beck's *Medical Jurisprudence*, Ray's *Treatise on Insanity*, and Warren's *Observations on Tumors*.

The third point to which attention is directed is "The Influence which is exerted upon American Medical Literature, by the American Medical Press," and upon this subject the report speaks with that boldness which truth and patrotism inspire, albeit the facts therein stated fall like melted iron upon that class of namby-pamby writers, whose lucubrations sometimes appear in our medical journals under the title of "Reviews." It is more particularly in reference to this branch of journalism that the author insists upon an improvement. The department of original communications is admitted to be fairly supported, and to abound in articles which will compare favorably, *in a practical point of view*, with similar articles in foreign periodicals, but it is here plainly, and, we dare say, truthfully asserted, that the department of criticism is sadly behind that of the British press in point of boldness, force, and good judgment.

"The reviews are, with few exceptions, written without taste and without point, as if their authors were afraid lest they should be accused of unkindness, harshness, or ill-nature. They are characterized more by politeness than by a manly and independent tone, which is not afraid to utter its real sentiments and to affix the seal of its unbiassed judgment. They are marked by none of the masculine vigor which is so well calculated to impart zest to the reader, and cause him to regret that he is so near the end of his task; which infuses life and spirit into a journal, and makes it a welcome guest at the table of the physician; which fashions and directs the dart, but blunts its point before it is permitted to strike its victim; which metes out equal justice to all men who come within its vitalizing, soul-stirring influence; which blends mercy with severity; which, when occasion requires, wounds but does not kill. It is a criticism which is neither alkaline nor acid, nor yet wholly neutral, but so nearly neutral as to render it impossible to determine its real character. It is a jesting, good-natured criticism, which, for fear of doing mischief, or of being thought unkind, is bound in swaddling-clothes, lest, by its sudden and inadvertent jerks, it should kick over the milk and water in the inkstand of the happy, self-complacent reviewer. In fine, it is an inert, a tame, a spiritless criticism; a criticism without body, without strength, without soul, deaf and dumb, and blind and halt."

The report then proceeds to state the essential elements of true criticism, and its importance to the profession, in the following terms.

"It should be free and independent of all extraneous influence, foreign and domestic. It should exercise its functions openly, boldly, vigorously; with an eye single to the honor of the profession and the glory of America. While it should ever be ready to rebuke egotism, presumption, and ignorance, however exhibited, or from whatever source emanating, it should also be ready to speak the word of gentleness and kindness to the brother who adds his feeble mite to the general stock of knowledge and experience. While it does not cover, as with a mantle, his sins of omission and commission, it should endeavor to point out his defects in the spirit of affection and encouragement. It should hail his efforts as a good omen, as an evidence of his zeal and devotion to the cause of science, as a desire to render himself useful, and not to bury his talent in the earth, and lead the life of a drone. It should encourage him to proceed, to renew his efforts, to try again. It should not aim to extinguish him by the discharge of its gall, by harsh rebuke, insolent assertion, or, worse than all, by faint praise, which has so often blasted the aspirations of genius, and damped the energies of mediocrity. Nor should it indulge in undue severity against the works of our European brethren, but extend to them a cordial, a hearty, a threefold welcome. It should, in the discharge of its noble mission, institute a thorough examination into their merits, and award them praise or censure, according to the dictates of its honest convictions. It should truckle to no man, clique, or faction; it should be subservient to no interests, save those of truth and justice. It should be patriotic, and at the same time cosmopolitan; local, and at the same time universal; for time, and at the same time for eternity. Like the Judge upon the bench, it should, in all doubtful cases, lean to the side of mercy, and never condemn without adequate testimony. It should be blind, yet far-sighted; mild, yet stern and uncompromising, watching, as with an eagle's eye, the honor, dignity, and interests of American medicine. Such, I conceive, is the criticism which should animate and characterize the medical press of the country. Any other than this is illiberal, and unworthy of its high mission."

One of the probable causes by which editors are more or less influenced, and a free expression of opinion more or less trammelled, is stated to be found in the obligation under which publishers place editors by presenting them with books to be reviewed. This is a very grave and serious charge, and has already had the effect of stirring up the passions of those whose consciences convict them of the offence. But the accusation is evidently made with no intention of wounding the feelings of any, not even those editors who appear to consider themselves the special victims of the "Report," but for the purpose of calling the attention of journalists to the fact that, by receiving books as presents, they necessarily, although doubtless unconsciously, incur an obligation, which it is but natural they should endeavor to repay whenever they can do so without a most palpable violation of the truth. The correctness of this view is clearly recognized by certain well-known English journals, who advertise that they will not receive books sent to them gratuitously, but will purchase such as they wish to review. In proof of the fact that a favorable notice of their publications is expected by our publishers, the reporter refers to instances in which these gentlemen have ceased all intercourse with journals in consequence of condemnatory notices of some of their publications. So that in addition to the mere courtesy thus demanded of the journalist, it is shown that it is also his interest in some cases to admit nothing in his pages likely to offend the publisher. But how far this influence operates is left to the decision of each individual concerned. In disclaiming all intention to cast any insult upon either medical editors or publishers the reporter adds:

"I have merely alluded to what I believe to be a habit into which some of the journalists of the country have fallen unconsciously in regard to the subject under discussion, and which they are unconsciously perpetuating. Nor, on the other hand, would I accuse the respectable body of republishers of any wrong intentions in their efforts to procure from the periodical press favorable notices of their productions. It is their business, as it is their interest, to promote the circulation of the works which they reprint. It is their pursuit; they live and grow fat by it. The American medical profession owes these republishers an immense debt for affording them in a cheap and accessible form, the works of our trans-atlantic brethren; as they owe us an immense debt for purchasing these works, and promoting their circulation, by the influence which we wield through the press, in the lecture-room, and in our private intercourse with each other. Thus far the obligation is mutual. But here let it cease. Let our journalists procure these reprints at their own expense, and we predict that a healthier tone will soon become apparent in their critical notices and reviews."

Under the fourth head, a still more prominent cause of impediment to the advance of our national medical literature, is stated to be "the little use that is made of the advantages afforded by private and hospital practice." Of our grievous neglect, as practitioners of medicine and surgery, to record the daily results of our observations in private practice, every one who takes a medical journal must be more or less aware, but it will no doubt astonish some of our readers to be informed, as we are told here, that not a single treatise on any branch of medicine or surgery has ever been furnished to the profession as the result of hospital observation. The author estimates the number of sick and wounded that annually enjoy the benefits of our various hospitals, almshouses, and asylums, at 120,000.

"The physicians, surgeons, and accoucheurs who have charge of them must amount to several hundred, embracing a large share of the best medical talent and intelligence of the country; and yet what have these institutions done, what have these physicians, surgeons, and accoucheurs done for American medical science, American medical art, and American medical literature? Where are the trophies which they have brought from this great battle-field of disease and accident? Where are the legacies which they have bequeathed, or which they are ready to bequeath to their profession and their country? Can any one point to one solitary work, of any note or respectability, that has emanated from their pen, as the legitimate result and effect of the immense opportunities which they have thus enjoyed? Where are the treatises which they have furnished us on clinical medicine, clinical surgery, and clinical obstetrics; on fevers, on eruptive diseases, and on diseases of the digestive organs, the lungs, the heart, and the brain; on wounds, fractures, dislocations, injuries of the skull, tumors, aneurism, amputations, resections, and various other important subjects? Where are their works on pathological anatomy and animal chemistry? Echo, alas! echo, alas! answers, Where, where! We have accomplished, literally and absolutely, nothing in any of these particulars. Need we be surprised, then, when a recent English writer* exclaims: 'We may safely say there is no American school of medicine; whereas there is a French, a German, an Italian, and an English. Our transatlantic offspring reprint, translate, and pirate the medical works of other nations, but they produce little of their own. Their pathology is chiefly French; their therapeutics English.' Mortifying as such an accusation is, it is certainly not wholly destitute of truth."

"Of the 120,000 patients who, we have supposed, are annually admitted into the various hospitals, asylums, and other charitable institutions of the country, at least ten thousand die. The bodies of many of these are doubtless examined, but where are the records of the results? I am not aware that one solitary great and important paper on pathological anatomy has ever appeared in our medical journals from the pen of a hospital physician, surgeon, or accoucheur."

In conclusion, the Report makes an earnest and patriotic appeal to the profession

"To throw off the yoke which has so long galled and oppressed us; to declare ourselves, as far as our schools are concerned, free and independent of the literature of Europe, from whatever quarter emanating; to encourage and foster our own authors; and, finally, to make the best possible use of the means, private and public, which are at our disposal for the establishment of an original, a vigorous, and an independent national medical literature.

"In view of the speedy and successful accomplishment of these desirable ends, I beg leave to submit the following resolutions:—

"*Resolved*, That this Association earnestly and respectfully recommend, first, the universal adoption, whenever practicable, by our schools, of American works as text-books for their pupils; secondly, the discontinuance of the practice of editing foreign writings; thirdly, a more independent course of the medical periodical press towards foreign productions, and a more liberal one towards American; and, fourthly, a better and more efficient employment of the facts which are continually furnished by our public institutions for the elucidation of the nature

* In Ranking's Half-Yearly Abstract, No. 22, p. 305, 1855. Philada., 1856.

of diseases and accidents, and, indirectly, for the formation of an original, a vigorous, and an independent national medical literature.

"*Resolved*, That we venerate the writings of the great medical men, past and present, of our country, and that we consider them as an important element of our professional and national glory.

"*Resolved*. That we shall always hail with pleasure any useful or valuable works emanating from the English press, and that we shall always extend to them a cordial welcome as books of reference, to acquaint us with the progress of legitimate medicine abroad, and to enlighten us in regard to any new facts of which they may be the repositories."

---

